# RN486, a Bruton's Tyrosine Kinase inhibitor, antagonizes multidrug resistance in ABCG2-overexpressing cancer cells

**DOI:** 10.2478/jtim-2024-0011

**Published:** 2024-07-27

**Authors:** Xing-Duo Dong, Qisi Lu, Yi-Dong Li, Chao-Yun Cai, Qiu-Xu Teng, Zi-Ning Lei, Zeng-Hui Wei, Fan Yin, Leli Zeng, Zhe-Sheng Chen

**Affiliations:** Department of Pharmaceutical Sciences, College of Pharmacy and Health Sciences, St. John's University, Queens, NY 11439, USA; Department of Hematology, Foresea Life Insurance Guangzhou General Hospital, Guangzhou 515500, Guangdong Province, China; Guangdong Provincial Key Laboratory of Digestive Cancer Research, Digestive Diseases Center, Biobank, Precision Medicine Center, Scientific Research Center, The Seventh Affiliated Hospital of Sun Yat-Sen University, Shenzhen 518107, Guangdong Province, China; Department of Statistics, University of California at Irvine, Irvine, CA 92697, USA

**Keywords:** RN486, bruton's tyrosine kinase inhibitor, multidrug resistance, ATP-binding cassette transporter, ABCG2

## Abstract

**Background and Objectives:**

Overcoming ATP-binding cassette subfamily G member 2 (ABCG2)-mediated multidrug resistance (MDR) has attracted the attention of scientists because one of the critical factors resulting in MDR in cancer is the overexpression of ABCG2. RN486, a Bruton’s Tyrosine Kinase (BTK) inhibitor, was discovered to potentially reverse ABCB1-mediated MDR. However, there is still uncertainty about whether RN486 has a reversal off-target impact on ABCG2-mediated MDR.

**Methods:**

MTT assay was used to detect the reversal effect of RN486 on ABCG2-overexpressing cancer cells. The ABCG2 expression level and subcellular localization were examined by Western blotting and immunofluorescence. Drug accumulation and eflux assay and ATPase assay were performed to analyze the ABCG2 transporter function and ATPase activity. Molecular modeling predicted the binding between RN486 and ABCG2 protein.

**Results:**

Non-toxic concentrations of RN486 remarkably increased the sensitivity of ABCG2-overexpressing cancer cells to conventional anticancer drugs mitoxantrone and topotecan. The reversal mechanistic studies showed that RN486 elevated the drug accumulation because of reducing the eflux of ABCG2 substrate drug in ABCG2-overexpressing cancer cells. In addition, the inhibitory efect of RN486 on ABCG2-associated ATPase activity was also verified. Molecular docking study implied a strong binding afinity between RN486 and ABCG2 transporter. Meanwhile, the ABCG2 subcellular localization was not altered by the treatment of RN486, but the expression level of ABCG2 was down-regulated.

**Conclusions:**

Our studies propose that RN486 can antagonize ABCG2-mediated MDR in cancer cells via down-regulating the expression level of ABCG2 protein, reducing ATPase activity of ABCG2 transporter, and inhibiting the transporting function. RN486 could be potentially used in conjunction with chemotherapy to alleviate MDR mediated by ABCG2 in cancer.

## Introduction

At present, chemotherapy remains the mainstay of treatment for diverse cancers.^[1]^ However, the development of multidrug resistance (MDR) increases the viability of cancer cells and hinders the success of chemotherapy.^[2,3]^ MDR relates to the resistance of cancer cells to structurally and mechanistically irrelevant types of chemotherapeutic drugs,^[4,5]^ which contributes to the low effectiveness of these distinct classes of anticancer drugs. This widespread issue ultimately results in cancer relapse and worse prognosis of patients. In humans, the principal cause of MDR is the overexpression of some members of the ATP-binding cassette (ABC) transporter family, which leads to decreased drug accumulation because of increased active efflux of the anticancer drugs across the cell membrane.^[6]^

Active membrane transport proteins renowned as ABC transporters are widely distributed in both prokaryotes and eukaryotes and have a variety of activities. They utilize the energy generated by the hydrolysis of ATP to transport endogenous ligands or exogenous drugs out of cancer cells.^[4,7]^ At present, 49 human ABC proteins have been discovered in this large superfamily. Based on sequences similarities, ABC transporters are divided into 7 subfamilies, ranging from ABCA to ABCG.^[8]^ ABCB1 (P-glycoprotein, P-gp; MDR1), ABCC1 (multidrug resistance protein 1, MRP1), and ABCG2 (breast cancer resistance protein, BCRP; mitoxantrone resistance, MXR) are three main proteins that have been studied for many decades. Their structures, functions, regulatory factors, and their significance in MDR, have been extensively reported.^[9,10]^ The 72 kDa protein, ABCG2, which is the second member of the ABCG subfamily, is an isolated protein from multidrug-resistant cancer cells.^[11, 12, 13]^ As recognized to be one of the main mediators of MDR, the overexpression of ABCG2 reduces the accumulation of chemotherapeutic drugs in cells by increasing efflux activity, which in turn causes decreased drug efficacy.^[4,14, 15, 16]^ The reported chemotherapeutic drugs comprise mitoxantrone, afatinib, osimertinib, camptothecin derivatives 9-aminocamptothecin, topotecan, irinotecan, and SN-38.^[17, 18, 19, 20]^ Hence, surmounting MDR induced by overexpressed ABCG2 has become a hotspot. The combination therapy, co-administration of ABC transporter inhibitors and anticancer drugs, is a potential approach which has been studied extensively.^[21, 22, 23, 24]^ Among these inhibitors, TKIs are the main contributors, for example, ponatinib,^[25]^ icotinib,^[26]^ and linsitinib,^[27]^ have displayed a significant reversal effect on ABCG2-mediated MDR. These reports indicated TKIs could be promising inhibitors in overcoming MDR mediated by ABCG2. Moreover, lemon-derived extracellular vesicles nanodrugs and ABCG2-binding aptamers are among the novel approaches that have received significant attention in research.^[28,29]^

Bruton’s tyrosine kinase (BTK) is essential for the development of several B-cell malignancies. BTK is specifically and reversibly inhibited by RN486. As a preclinical drug, RN486 could effectively treat rheumatoid arthritis (RA).^[30,31]^ In addition, RN486 completely suppressed systemic lupus erythematosus (SLE) progression in a mouse model of spontaneous SLE.^[32]^ Our previous study revealed a significant off-target reversal effect of RN486 on ABCB1-meditated MDR.^[33]^ However, uncertainty persists on whether RN486 has the off-target reversal impact on ABCG2-mediated MDR. In this study, it was corroborated that RN486 could also antagonize the ABCG2-mediated MDR and restore the efficacy of ABCG2 substrate drugs.

## Materials and methods

### Chemicals

RN486 was generously provided by ChemieTek (Indianapolis, IN). Other chemicals such as mitoxantrone, topotecan, Ko 143, cisplatin, verapamil, and 4′, 6-diamidino-2-phenylindole (DAPI) were obtained from Sigma-Aldrich (St. Louis, MO). Fetal bovine serum, Dulbecco’s modified eagle’s medium, and 0.25% trypsin were sourced from Corning Inc. (Corning, NY). Human monoclonal antibody against ABCG2 was purchased from Millipore (Billerica, MA). Horseradish peroxidase (HRP)-conjugated rabbit anti-mouse IgG secondary antibody was acquired from Cell Signaling Technology Inc. (Danvers, MA). Loading control GAPDH and Alexa Fluor 488 conjugated goat anti-mouse IgG secondary antibodies were procured from Thermo Fisher Scientific Inc (Rockford, IL). [^3^H]-labeled mitoxantrone (2.5 Ci/mmol) was purchased from Moravek Biochemicals (Brea, CA). All other chemicals were obtained from Sigma Co (St. Louis, MO).

### Cell lines and cell culture

The parental non-small cell lung cancer (NSCLC) cell line NCI-H460, human colon adenocarcinoma cell line S1, and their mitoxantrone-selected ABCG2-overexpressing NCI-H460/MX20 cells and S1-M1–80 cells were used. Concurrently, human embryonic kidney HEK293 cells were used to establish HEK293/PEL, HEK293/B1, HEK293/ G2, and HEK293/B1G2 cell lines by transfecting of an empty vector, vectors encoding full-length human ABCB1 or ABCG2, and a bicistronic ABCB1/ABCG2 expression vector, respectively.^[34]^ Zeocin of 250 mg/mL was required to screen the transfected cells.^[34]^ All previous mentioned cells were cultured in the 37 ℃ and moist incubator with 5% CO_2_.

### Cytotoxicity and reversal assays

MTT assay was conducted as previously described.^[35]^ In 96-well plates, cells were evenly seeded (5, 000 cells/well) and incubated overnight. For cytotoxicity experiments, the RN486 with gradient concentrations (0–100 μM) was added into designated wells. For reversal experiments, RN486 with distinct concentrations (0.3, 1, or 3 μM), positive control ABCB1 inhibitor verapamil (3 μM), or positive control ABCG2 inhibitor Ko143 (3 μM) were applied 2 h in advance of adding the diluted conventional anticancer drug. The plates were then incubated for 68 h, followed by an additional 4 h incubation with 4 mg/mL MTT solution. Afterward, the medium was substituted with 100 μL of DMSO. Once the plates were shaken, formazan crystals dissolved completely. At 570 nm, absorbance was recorded using a UV/Vis Microplate Spectrophotometer. The half-maximal inhibitory concentration (IC_50_) for each treatment was calculated.

### Western blotting

NCI-H460 and NCI-H460/MX20 cells were incubated with or without the optimal concentration of RN486 (3 μM) up to 72 h. Then, the cell lysis, protein quantification, gel electrophoresis, and Western blotting studies were performed in accordance with previously reported protocols.^[36,37]^ ImageJ was used for quantitative analysis.

### Immunofluorescence assay

The subcellular location of ABCG2 was confirmed using an immunofluorescence assay, as previously described.^[38]^ In brief, NCI-H460 and NCI-H460/MX20 cells were seeded into 24-well plates and cultured with or without RN486 (3 μM) up to 72 h. After cells were fixed (4% formaldehyde) and permeabilized (0.25% Triton X-100), BSA (6%) blocking was carried out. Following this, ABCG2 primary and Alexa Fluor 488 conjugated IgG secondary antibodies (1: 1000) were applied. DAPI solution was employed to counterstain the nuclei. Using a Nikon TE-2000S microscope, fluorescence images were taken.

### Drug accumulation and efflux assay

[^3^H]-Mitoxantrone was used in accumulation and efflux assays, as previously reported.^[39,40]^ In short, equal density (1 × 10^5^ cells/well) of NCI-H460 and NCI-H460/ MX20 cells were seeded in 24-well plates. After overnight incubation, RN486 (0, 1, and 3 μM) or Ko143 (3 μM) were added to the assigned wells 2 h earlier than treating with [^3^H]-mitoxantrone. Following 2 h of incubation with [^3^H]-mitoxantrone, the medium was aspirated and substituted with the only medium comprising the reversal reagent. At different time points (0, 30, 60, and 120 min), cells were washed, lysed, and collected into vials containing 5 mL of scintillation liquid. A scintillation analyzer from Packard Instrument Company, Inc (Downers Grove, IL, United States) was utilized to examine the radioactivity of each treatment.

### ATPase assay

PREDEASY ATPase assay kit (TEBU-BIO nv, Boechout, Belgium) was used to detect the ABCG2-associated ATPase activity, as previously described.^[33]^

### Molecular modeling

Molecular modeling was carried out utilizing Maestro v12.6 software (SchrÖdinger, LLC, New York, NY, 2020). The human ABCG2 protein (PDB ID: 6FFC) was prepared,^[41]^ and a grid was produced using default setting. The ligand was constructed and ready for Glide XP docking. Subsequently, the top-ranked docking scores were acquired with default configuration.^[42]^

### Statistical analysis

Data shown as mean ± SD were produced from at least three independent experiments. One-way ANOVA and post hoc Tukey’s test were conducted to evaluate the difference among various groups, and a P < 0.05 was considered statistical significance.

## Results

### RN486 reverses ABCG2-mediated MDR in ABCG2-overexpressing cancer cells

**Figure 1:**
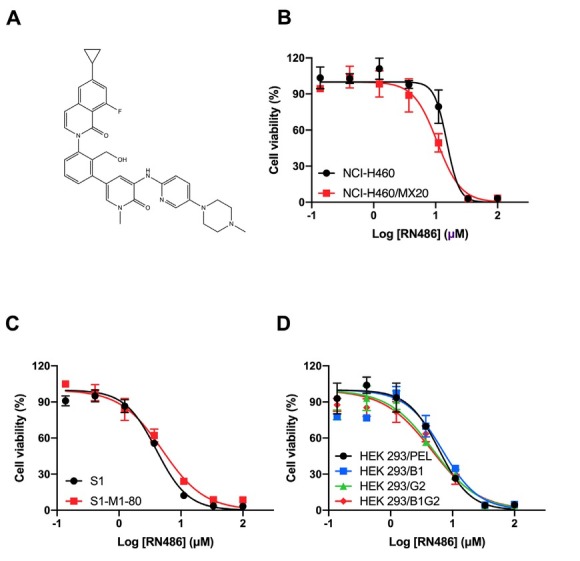
The cytotoxicity of RN486 in ABCG2 overexpressing cancer cells and HEK293 transfected cells. (A) The description of RN486’s chemical composition. (B-C) The cytotoxicity of RN486 on NCI-H460 and NCI-H460/MX20, S1 and S1-M1-80 cells. (D) Cytotoxic effect of RN486 on HEK293/PEL, HEK293/B1, HEK293/ G2, HEK293/B1G2 cells. As a delegate of three separate experiments, the data are provided as mean ± SD.

**Figure 2:**
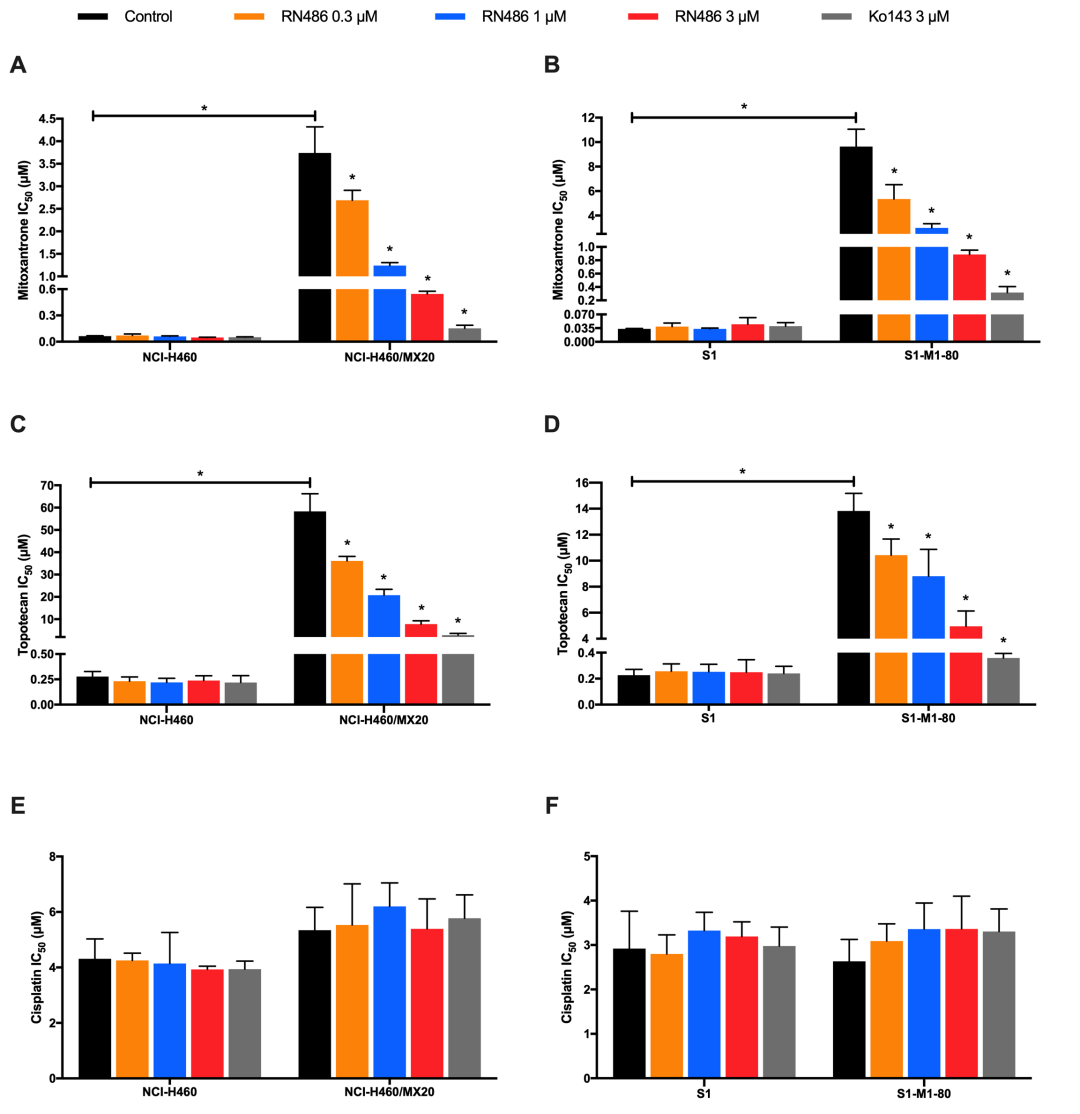
The effect of RN486 on the cytotoxicity of anticancer drugs in ABCG2-overexpressing cancer cells. RN486 affected the cytotoxicity of mitoxantrone (A), topotecan (C), and cisplatin (E) in NCI-H460 and NCI-H460/MX20 cells. The cytotoxicity of mitoxantrone (B), topotecan (D), and cisplatin (F) in S1 and S1-M1-80 cells when co-treated with various concentrations of RN486. Ko143 (3 μM) served as a positive control. As a delegate of three separate experiments, the data are provided as mean ± SD. In comparison to the pertinent control group, *P < 0.05.

**Figure 3:**
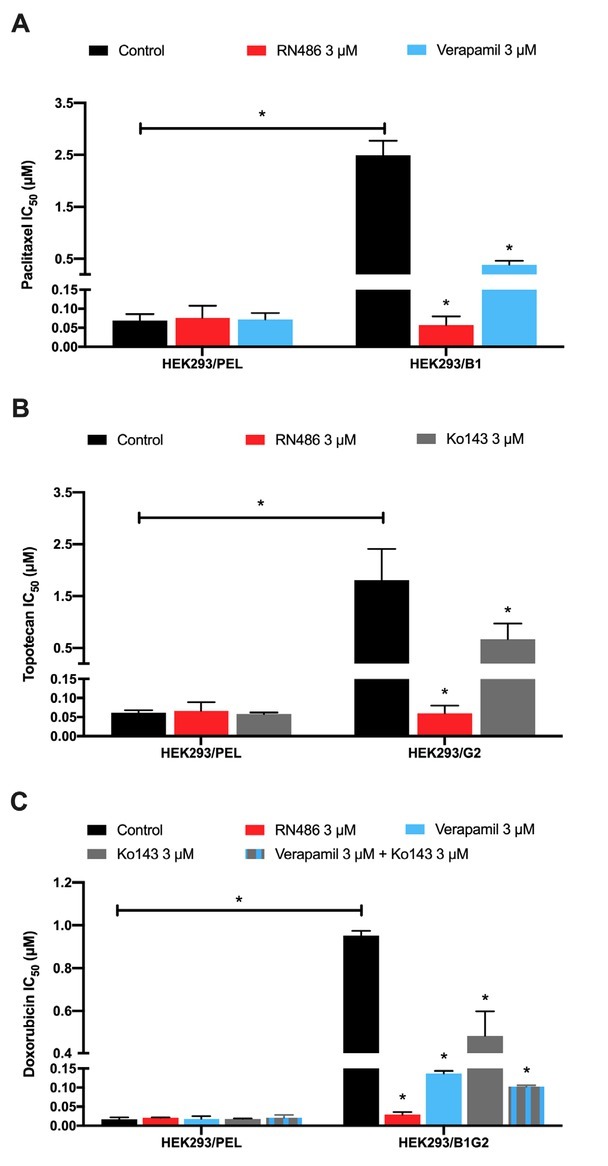
The effect of RN486 on the cytotoxicity of anticancer drugs in HEK293 transfected cells. RN486 affected the cytotoxicity of paclitaxel (A) in HEK293/ PEL and HEK293/B1 cells, topotecan (B) in HEK293/PEL and HEK293/G2 cells, and doxorubicin (C) in HEK293/PEL and HEK293/B1G2 cells. Verapamil (3 μM) and Ko143 (3 μM) served as positive controls. As a delegate of three separate experiments, the data are provided as mean ± SD. In comparison to the pertinent control group, *P < 0.05.

The structure of RN486 was exhibited in Figure 1A. MTT assay was performed firstly to determine the cytotoxicity of RN486 in NSCLC, colon cancer, and HEK293 cell lines (Figure 1B-D). Non-toxic concentrations of RN486 (0.3, 1, and 3 μM) were opted in further reversal experiments. As displayed in Figure 2A-D, the drug-resistant NCI-H460/ MX20 and S1-M1–80 cells possessed much higher IC_50_ values than parental NCI-H460 and S1 cells when treated with traditional anticancer drugs mitoxantrone or topotecan. The combination strategy of these anticancer drugs with RN486 or positive reversal reagent Ko143 showed an excellent re-sensitization effect on the drug-resistant cell lines. Additionally, this reversal proceeded in an RN486 concentration-dependent manner. The IC_50_ values of mitoxantrone in NCI-H460/MX20 and S1-M1–80 were decreased strikingly by 3 μM RN486 (Figure 2A-B). Similarly, the RN486 at 3 μM was able to dramatically decrease the IC_50_ values of topotecan in NCI-H460/MX20 and S1-M1–80 cell lines (Figure 2C-D). However, no significant difference was found between various treatment groups in both parental and drug-resistant cell lines when we used the non-ABCG2-substrate drug cisplatin (Figure 2E-F). Further verification of the reversal effect of RN486 was conducted on a set of transfected HEK293 cell lines. The outstanding reversal effect of RN486 on HEK293/B1 cells was consistent with the previous report (Figure 3A).^[33]^ RN486 (3 μM) significantly reduced the IC_50_ of topotecan in HEK293/ G2 cells as shown in Figure 3B. In HEK293/B1G2 cells, RN486 produced stronger reversal ability than Ko143 or verapamil alone as well as the combination of verapamil (3 μM) with Ko143 (3 μM) (Figure 3C). Verapamil, a well-known ABCB1 inhibitor, acted as a positive control reversal agent. It should be noted that doxorubicin used in HEK293/B1G2 cells was the substrate of both ABCB1 and ABCG2 transporters.

### RN486 down-regulates ABCG2 expression and does not interfere subcellular localization of ABCG2 in ABCG2-overexpressing cancer cells

**Figure 4:**
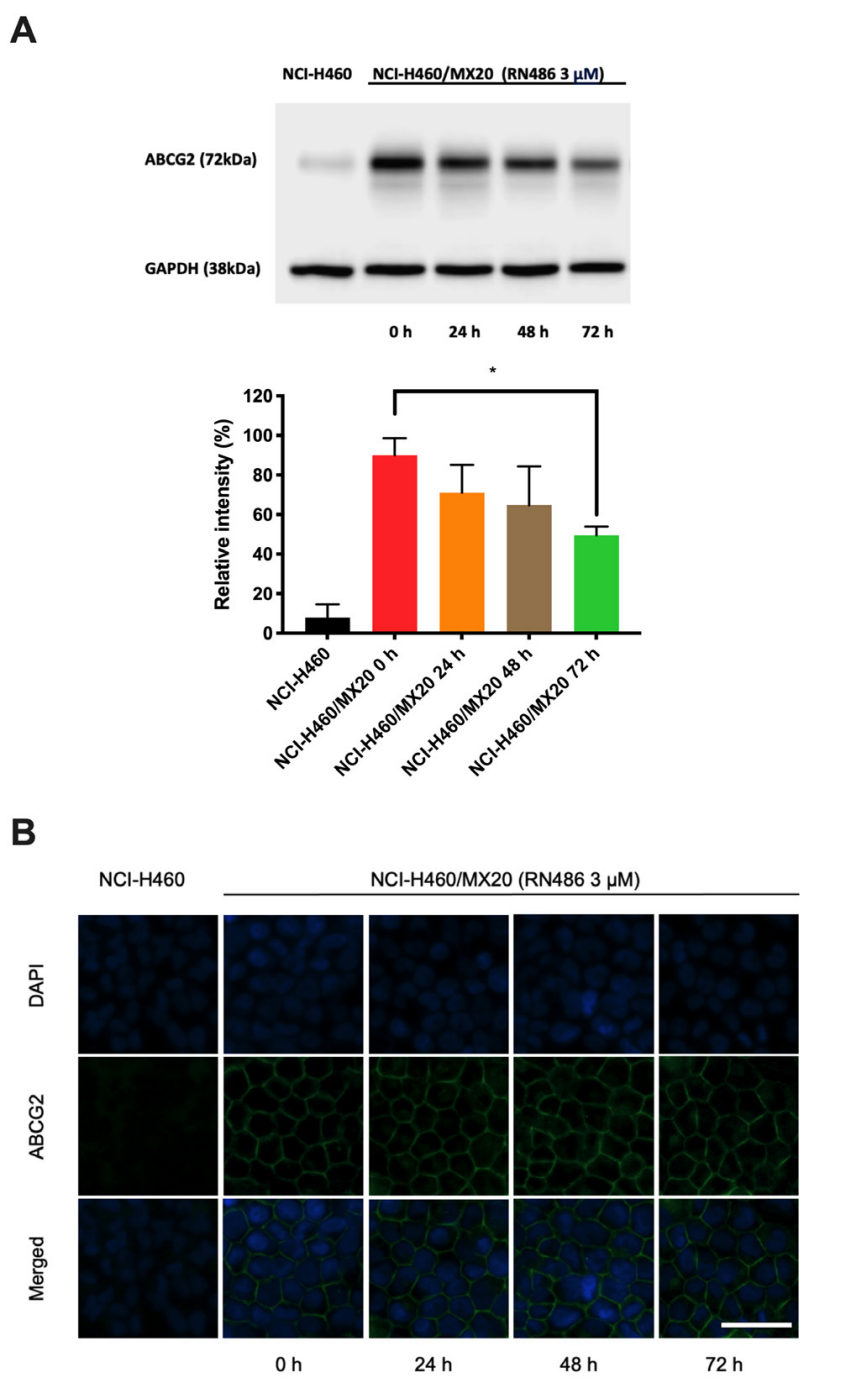
The effect of RN486 on the protein expression level and localization of ABCG2. (A) The ABCG2 expression level treating with 3 μM of RN486 for 0, 24, 48, and 72 h in NCI-H460/MX20 cells as well as the relative intensity of each treatment. (B) ABCG2 transporter immunofluorescence of the subcellular localization after treatment with 3 μM of RN486 for 0, 24, 48, and 72 h. NCI-H460 acts as the control group. Scale bar, 25 μm. As a delegate of three separate experiments, the data are provided as mean ± SD. *P < 0.05 versus the relevant control group.

It is acknowledged that altered ABCG2 expression levels and modified ABCG2 positioning are possible mechanisms for the abatement of MDR by reversal reagents. Immunoblotting and immunofluorescence were performed to examine the changes of ABCG2 protein that may have occurred in ABCG2-overexpressing cells treated with RN486. The appropriate concentration of RN486 (3 μM) was used. As Figure 4A revealed, the expression level of ABCG2 diminished notably by RN486 in a time-dependent manner in NCI-H460/MX20 cells. Meanwhile, RN486 (3 μM) did not possess the ability to change the localization of ABCG2 in NCI-H460/MX20 cells (Figure 4B). Our findings suggested that the down-regulation of ABCG2 contributes to the reversal effect of RN486 and the potential of altering ABCG2’s subcellular location was eliminated.

### RN486 boosts [^3^H]-mitoxantrone accumulation in ABCG2-overexpressing cells

**Figure 5:**
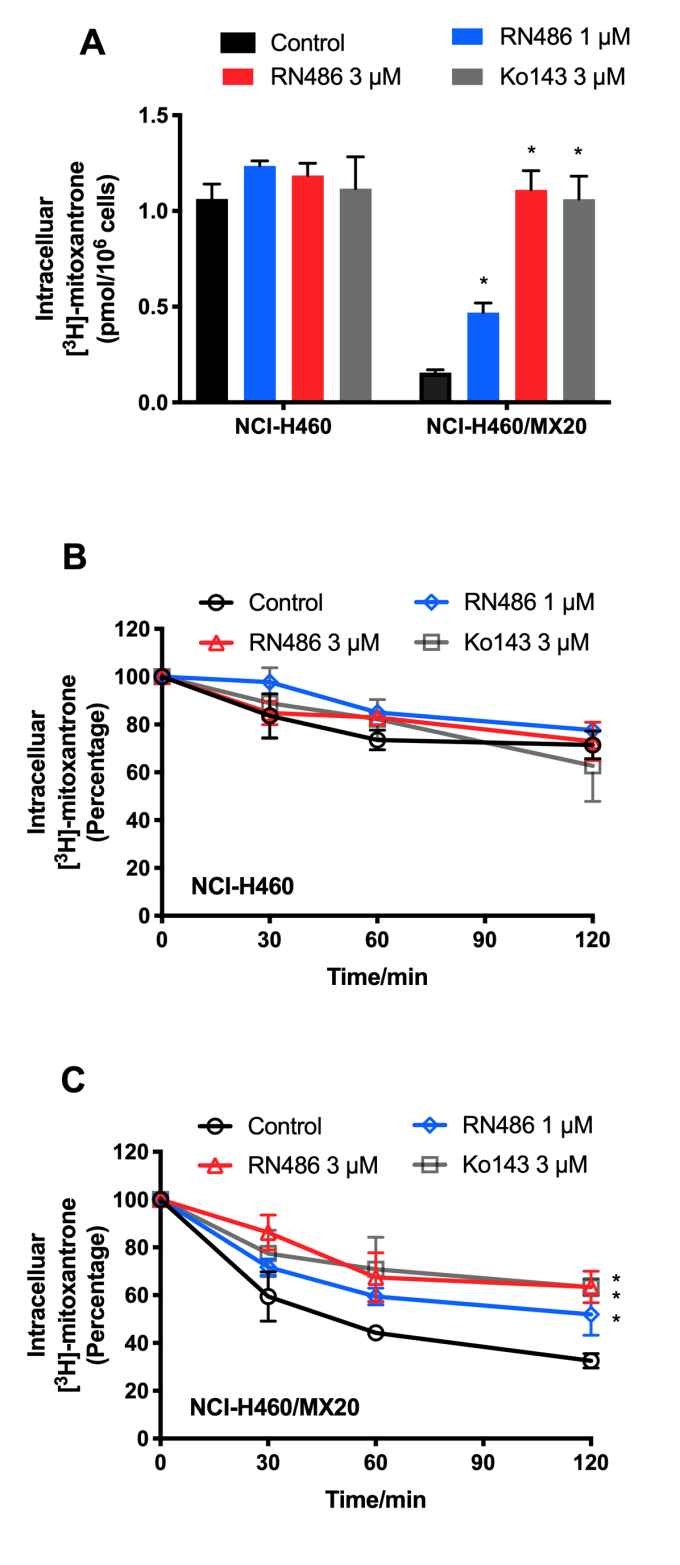
RN486 increased the intracellular [3H]-drug accumulation and inhibited the efflux of ABCG2 transporter in cancer cells overexpressed ABCG2. (A) The effect of RN486 on the accumulation function of [3H]-mitoxantrone in ABCG2-mediated MDR cell lines. (B-C) The effects of RN486 on the efflux of [3H]-mitoxantrone in NCI-H460 and NCI-H460/MX20 cells. Ko143 (3 μM) served as a positive control. As a delegate of three separate experiments, the data are provided as mean ± SD. *P < 0.05 versus the relevant control group.

The results above indicated that the down-regulation of ABCG2 was confirmed as one contributor to the reversal effect of RN486. In order to gain a deeper comprehension of reversal mechanism, we have shifted our focus towards examining the transporter’s function. The intracellular [^3^H]-mitoxantrone levels were prominently increased with the treatment of 1 μM and 3 μM RN486 in drug-resistant NCI-H460/MX20 cells compared to the control group (Figure 5A). Moreover, RN486 has a comparable effect of the positive control Ko143 when incubated with the same concentration. However, in parental NCI-H460 cells, no significant difference was shown between distinct groups. These findings suggested that RN486 participates in mediating the function of the ABCG2 transporter, which results in the enhanced accumulation of [^3^H]-mitoxantrone.

### RN486 attenuates [^3^H]-mitoxantrone efflux in ABCG2-overexpressing cells

Following the accumulation assay, an efflux assay was performed to evaluate the effect of RN486 on the efflux activity of ABCG2. As Figure 5C displayed, the higher concentration of RN486 (3 μM) remarkably decreased the efflux of [^3^H]-mitoxantrone similar to Ko143 (3 μM) in ABCG2-overexpressing cells, however, RN486 hindered the efflux process to a lower extent at 1 μM. For distinct treatments in parental cells, no significant difference was observed (Figure 5B). These results illustrated the inhibitory effect of RN486 on the efflux of the anticancer drug from the ABCG2 transporter.

### RN486 inhibits ATPase activity of ABCG2

**Figure 6:**
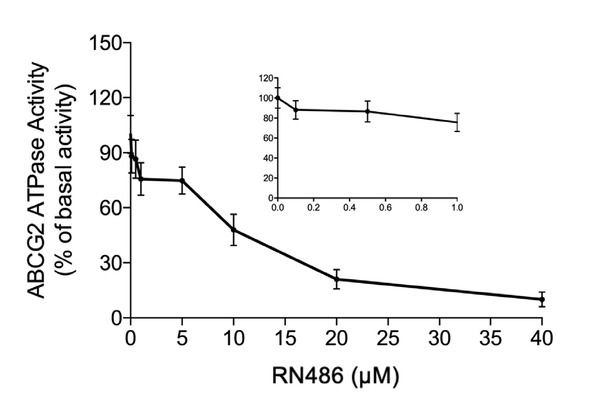
The effect of RN486 on the ATPase activity of ABCG2. The ATP hydrolysis process was impacted by RN486 at various concentrations (0–40 M), which was mediated by ABCG2 ATPase. The graph inset shows how RN486 in concentrations between 0 and 10 M affects ABCG2’s ATPase activity.

The ATP hydrolysis activity was considered as one of the most pivotal factors affecting the function of ABC transporters. This process provides the necessary energy for the transporters to actively transport substrate drugs across the membrane. The basal ATPase activity of ABCG2 was further investigated with the presence of RN486 (0–40 μM). In Figure 6, there was an observable decrease in ATPase activity, with a minimum plateau of 10%. Additionally, the inhibitory effect of RN486 on ABCG2 was found to reach half-maximum (EC_50_) at a concentration of 12 μM.

### Docking analysis of RN486 with ABCG2 protein

**Figure 7:**
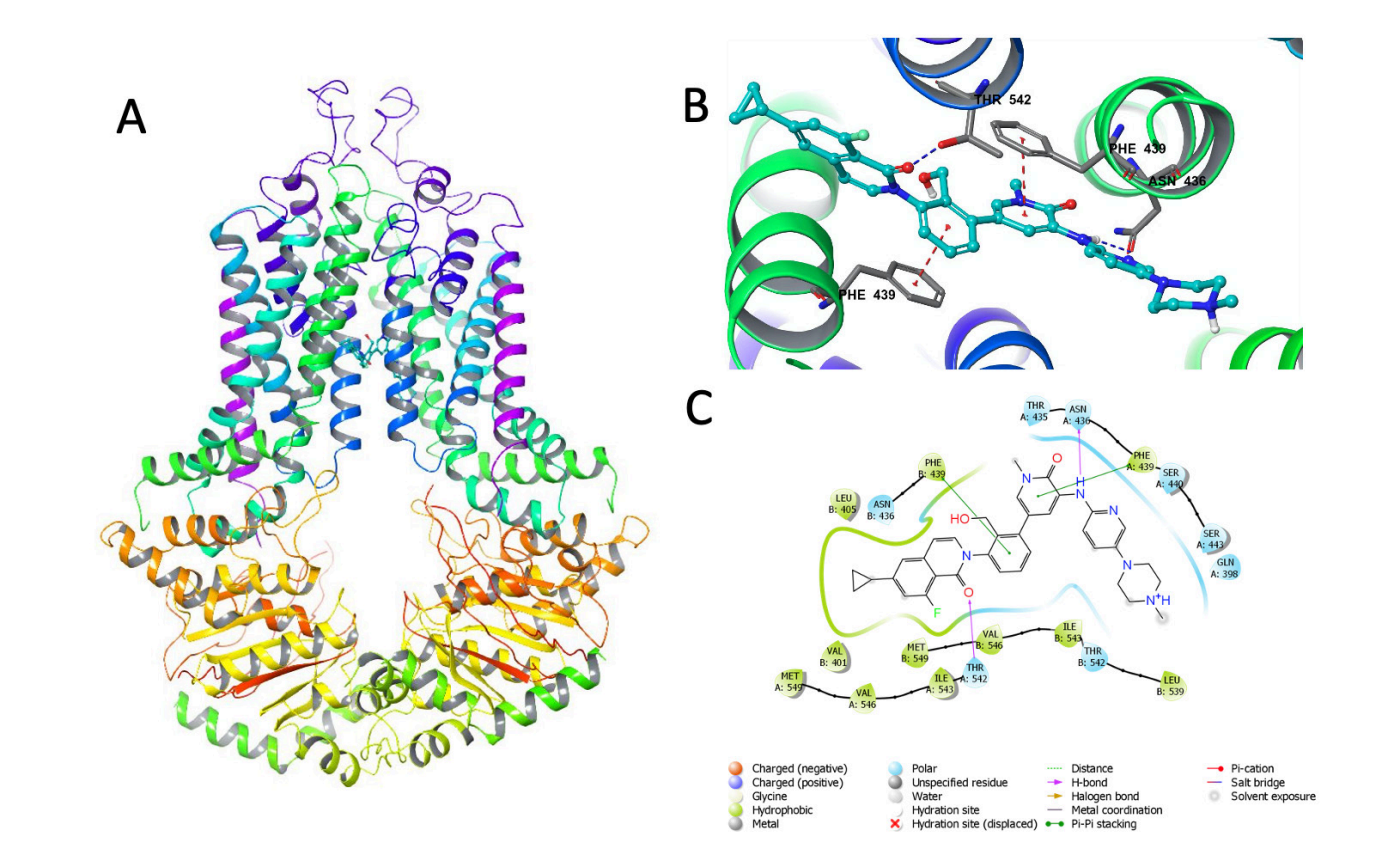
Investigating the relationship between RN486 and the human ABCG2 transporter protein. (A) A general depiction of RN486’s association with ABCG2 protein. (B) RN486 positioned within the human ABCG2 transporter protein. RN486’s atoms are colored accordingly: hydrogen- white, carbon- cyan, nitrogen-blue, fluorine- green, and oxygen- red. Pi stacking is shown by red dotted lines. Blue dashed lines represent hydrogen bonds. (C) Key interactions between RN486 and the binding site residues of human ABCG2 are displayed in a 2D schematic.

The docking outcomes revealed that RN486 interacted with the human ABCG2 protein, obtaining a high docking score of-13.183 kcal/mol, which signifies robust binding affinity between RN486 and the ABCG2 protein. Figure 7 displays the docking specifics. The oxopyridine group and phenyl ring of RN486 engaged in π-π interaction with Phe439 on the different chains of ABCG2 model, respectively. The amino group of RN486 constructed a hydrogen bond with Asn436 on the side chain of ABCG2. The isoquinolin ring’s carbonyl group in RN486 created a hydrogen bond with the Thr542 residue of ABCG2 protein. Furthermore, RN486 interacted with ABCG2 residues, such as Gln398, Asn436, Thr435, Ser440, Ser443, and Thr542 via hydrophilic effects, and Val401, Leu405, Leu539, Ile543, Val546, and Met549 through hydrophobic effects.

## Discussion

MDR continues to pose a significant challenge to successful cancer treatment and is now recognized as one of the major factors contributing to treatment failure.^[3,43]^ The ABC transporter positioned at the cell membrane restricts the bioavailability of delivered drugs. In cancer cells, the overexpression of ABC transporters impaired the efflux of chemotherapeutic drugs, which finally weakened the drug efficacy. To address this sticky issue, numerous research has been implemented. It turns out that combination therapy, which involves a chemotherapeutic drug with a reversal reagent, is the prospective approach to regulate the aberrant function of ABC transporters.^[4]^ TKIs used for certain cancer treatments generally have “off-target” impacts.^[44,45]^ In past decades, TKIs exhibit potential re-sensitization effects to overcome ABC transporter-mediated MDR in combination with other chemotherapeutic drugs.^[46]^ Hence, the usage of TKIs to restore the effect of traditional anticancer drugs is regarded as an alternative therapy in ABC transporter-associated MDR. RN486 is a selective, and reversible inhibitor targeting BTK. It has been reported that RN486 could potentially inhibit glycoprotein VI-mediated platelet activation and the ensuing amplification of inflammation caused by the platelet microparticles-induced generation of fibroblast-like synoviocytes cytokines.^[47]^ Besides, a recent study demonstrated that RN486 effectively enhances the efficacy of standard-of-care chemotherapy and EGFR-targeted therapy in drug-resistant NSCLC cell lines.^[48]^ Notably, our prior research showed that RN486 significantly reversed ABCB 1-meditated MDR.^[33]^ Here, we reported for the first time that RN486 could also conquer MDR in cancer cell lines with overexpressed ABCG2 transporter.

In this study, the cytotoxicity of RN486 was first assessed in both drug-induced and gene transfected cell lines to eliminate the probable bias in reversal experiments by MTT assay. The non-toxic concentrations of RN486 (0.3, 1, and 3 μM) were the optimal setting and were used in the following reversal studies. Our findings suggested that the desensitization of NCI-H460/MX20 and S1-M1–80 cells to mitoxantrone and topotecan was significantly diminished in the presence of RN486. However, RN486 did not affect the sensitivity of mitoxantrone and topotecan in NCI-H460 and S1 cells. Since drug-selected MDR was complex and may contain miscellaneous mechanisms but the transfected subset did not, the reversal effect of RN486 on ABCG2-overexpressing cells was further confirmed in ABCG2-transfected HEK293/G2 cells. Likewise, RN486 revealed a strong ability to decrease the resistance of topotecan in HEK293/G2 cells. Moreover, the potential reversal effect of RN486 on HEK293/B1 cells was also certified. Given that RN486 could overcome both ABCB 1-and ABCG2-mediated MDR, we hypothesized that it could exhibit stronger reversal effects than inhibitors of single ABC transporter in cells co-expressing ABCB1 and ABCG2 transporters. As expected, the IC_50_ of doxorubicin was remarkably lowered with the addition of RN486 in HEK293/B1G2, whereas only a partial reversal effect was observed in the treatment with a single inhibitor, Ko143 or verapamil. Surprisingly, the reversal capacity of RN486 surpassed that of the group containing Ko143 and verapamil, the two positive controls. The reversal effect of RN486 on cancer cells co-overexpressing ABCB1 and ABCG2 may be advantageous in the case of ABCB1 and ABCG2 co-overexpressed cells, given that when cancer cells co-overexpress multiple transporters, the inhibitor of one transporter may be the substrate of other transporters, resulting in weakened reversal effect.^[34]^ Furthermore, no discernible impact was found in cancer cells when incubated with cisplatin, which was not a substrate for either ABCB1 or ABCG2. Together, the MTT data illustrated the potential capability of RN486 on surmounting ABCG2-related MDR and patients suffering from both ABCB1 and ABCG2 overexpression may benefit from using RN486 in conjunction with conventional chemotherapeutic agents.

To uncover the reversal mechanism of RN486, we further evaluated the expression level and translocation condition of ABCG2 after treating ABCG2-overexpressing cells with RN486 for up to 72 h by performing Western blotting and immunofluorescence assay. After treatments, the ABCG2 expression level was apparently attenuated in an RN486 time-dependent way. As a crucial modulator of B cell receptor (BCR) signaling and then adaptive immunity, BTK has received extensive characterization.^[49]^ Numerous non-receptor tyrosine kinases, including Src, Jak, Syk, and FAK families, modulated BTK. BTK as well regulated various critical signaling pathways, such as PI3K, PLC, and PKC.^[50]^ Consequently, further research into whether probable alterations of upstream and downstream signals may regulate the expression of ABCG2 is necessary. No significant variation in subcellular localization was observed after treatment with RN486 (3 μM) for up to 72 h, implying that the change in the subcellular localization of the ABCG2 was not responsible for the reversal of the effect of RN486 on MDR. Additionally, more in-depth investigations should be undertaken for scientific research, such as the use of higher concentrations and longer treatment periods.

Afterwards, [^3^H]-mitoxantrone was used to determine the RN486’s impact on the function of ABCG2 transporter. According to our research, RN486 prominently elevated the intracellular [^3^H]-mitoxantrone concentration in ABCG2-overexpressing NCI-H460/MX20 cells, whereas no significant [^3^H]-mitoxantrone difference was observed in the respective parental cells. Besides, the efflux of [^3^H]-mitoxantrone from cells overexpressed ABCG2 transporter was considerably diminished in the presence of RN486, but not from the related parental cells. These [^3^H]-mitoxantrone accumulation and efflux results were in accordance with the reversal study, elaborating that RN486 weakened the pumping function of ABCG2 transporter, ultimately causing enhanced accumulation of the substrate-drug in drug-resistant cells and restoration of the chemotherapeutic efficacy.

Another non-negligible factor that influences the ABCG2 transporter is its ATPase activity, as the ATP is essential for the ABC transporter to function. Certain ABCG2 substrates or inhibitors can stimulate or inhibit the ABCG2 ATPase, which would either facilitate the pumping or impair the drug efflux from the intracellular to the extracellular side, depending on the case. As our results showed, RN486 evidently suppressed the ATPase activity in a concentration-dependent manner, achieving a minimum plateau of 10%.

The molecular docking analysis is one of the most commonly used techniques to anticipate the interaction between the proposed ligand and the protein as it simulates the potential binding affinity. Therefore, we designed the docking study to comprehend how RN486 interacts with ABCG2 transporter. In so doing, RN486-ABCG2 complex was stabilized by the π-π interaction, hydrogen bonding, hydrophilic and hydrophobic impacts of RN486 on ABCG2, which resulted in the greatest affinity score of-13.183 kcal/mol. This high-affinity score demonstrated the intense association of RN486 with ABCG2 protein. Although the mimic binding of RN486 to ABCG2 further supports our findings, the docking study still needs more validation. The ATPase and docking analysis results together also suggested that in addition to down-regulating of ABCG2 protein, RN486 probably interacts directly with the ABCG2 transporter to exhibit the functional deficiency.

To date, clinical usage of chemotherapeutic drugs still encounters therapy failure due to MDR. Among the primary causes of MDR, overexpression of ABC transporters in cancers diminished the remission of chemotherapy.^[51]^ The gradually increased number of anticancer drugs were identified as the substrates of ABCG2 and overexpression of ABCG2 was recognized to be closely related to the resistance of these drugs, necessitating the development of a novel treatment for ABCG2-mediated MDR.^[52,53]^

In conclusion, our study showed that RN486 can reverse MDR caused by ABCG2 overexpression through down-regulating the expression level of ABCG2 protein, inhibiting the efflux function of ABCG2 transporter, and restricting ATPase activity of ABCG2 transporter. In addition, the comparison of just RN486 treatment with co-treatment of verapamil and Ko143 in HEK293/ B1G2 cells reversal experiments illustrated the potentially excellent reversal effect of RN486. Clinical patients typically have multiple ABC transporters overexpressed, such as in patients with acute myeloid leukemias, rendering the use of reversal agents that exclusively target a single ABC transporter ineffective. Despite the complexity of the tumor microenvironment, which requires further confirmation including in vivo study, this work revealed a new approach of combining RN486 with conventional or innovative anticancer drugs for cancer patients diagnosed with overexpressed ABCB1 and ABCG2.
